# Supplementary material for: A multi-ethnic meta-analysis identifies novel genes, including ACSL5, associated with amyotrophic lateral sclerosis
Source: Commun Biol. 2020 Sep 23;3:526. doi: 10.1038/s42003-020-01251-2 (PMC7511394; doi:10.1038/s42003-020-01251-2)
Supplement: Supplementary file 1 — Supplementary Information [file 42003_2020_1251_MOESM1_ESM.pdf]

A multi-ethnic meta-analysis identifies novel genes, including *ACSL5*, associated with amyotrophic lateral sclerosis

Content:

Supplementary Note

- Members of the Japanese Consortium for Amyotrophic Lateral Sclerosis research (JaCALS)

Supplementary Figures

- Supplementary Figure 1. The genotyping and imputation workflow for Japanese ALS patients and controls.
- Supplementary Figure 2. Manhattan plot of the GWAS results of a Japanese (JaCALS) cohort.
- Supplementary Figure 3. Q–Q plots of the association –log10(P) between SNPs and ALS by GWAS of a Japanese (JaCALS) cohort.
- Supplementary Figure 4. Forest plots showing the effects of rs58854276 and rs11195948 in *ACSL5* in each cohort and meta-analysis
- Supplementary Figure 5. Box plot of *ACSL5* mRNA expression between sporadic ALS and controls.

Supplementary Tables

- Supplementary Table 1. Clinical characteristics of the patients used for the gene expression analysis of *ACSL5*.
- Supplementary Table 2. Primer sequences for quantitative real-time PCR

Supplementary Datas

- Supplementary Data 1. Summary of the SNPs with a suggestive threshold of $p = 5 \times 10^{-6}$ in the GWAS of a Japanese (JaCALS) cohort.
- Supplementary Data 2. Summary of the SNPs with a suggestive threshold of $p = 5 \times 10^{-6}$ in the GWAS of the meta-analysis between European and Japanese (JaCALS) cohorts.
- Supplementary Data 3. Summary of the SNPs with a significant threshold of $p = 5 \times 10^{-8}$ in the GWAS study of the meta-analysis among European, Japanese (JaCALS), and Chinese cohorts.
- Supplementary Data 4. The data of expression of *ACSL5* mRNA in LCLs from ALS patients with each genotype of rs3736947.
- Supplementary Data 5. Summary of genes with a suggestive threshold of $p = 2.85 \times 10^{-4}$ in the multi-ethnic meta-analysis of gene-based association analysis among European, Japanese (JaCALS), and Chinese cohorts.

Members of the Japanese Consortium for Amyotrophic Lateral Sclerosis research (JaCALS)

JaCALS members included Drs Tatsuhiko Yuasa (Kamagaya General Hospital, member of the JaCALS steering committee); Tomoko Nakazato (Juntendo University); Yukio Fujita, Yoshio Ikeda (Gumma University); Tomokazu Obi (Shizuoka Institute of Epilepsy and Neurological Disorders); Hiroaki Ito (Miyagi National Hospital); Seiya Noda, Satoshi Kuru(National Hospital Organization Suzuka National Hospital); Ikuko Iwata (Hokkaido University); Akihiro Kawata (Tokyo Metropolitan Neurological Hospital); Tadashi Kanouchi, Takanori Yokota (Tokyo Medical and Dental University); Yasuhiro Watanabe (Tottori University); Hiroyuki Ishiura, Shoji Tsuji (Tokyo University); Yukiko Tsuji, Toshiki Mizuno (Kyoto Prefectural University); Takashi Ayaki, Ryosuke Takahashi (Kyoto University); Takuji Fujita (Takumi-kai Neurology Clinic); Hiroshi Doi, Hideyuki Takeuchi, Fumiaki Tanaka (Yokohama City University); Takamura Nagasaka, Yoshihisa Takiyama (Yamanashi University); Wataru Shiraishi, Jun-ichi Kira (Kyushu University); Hitoshi Aizawa (Tokyo Medical University); Mie Nakamura (National Hospital Organization Tokyo National Hospital); and Yuji Takahashi (National Center Hospital, National Center of Neurology and Psychiatry).

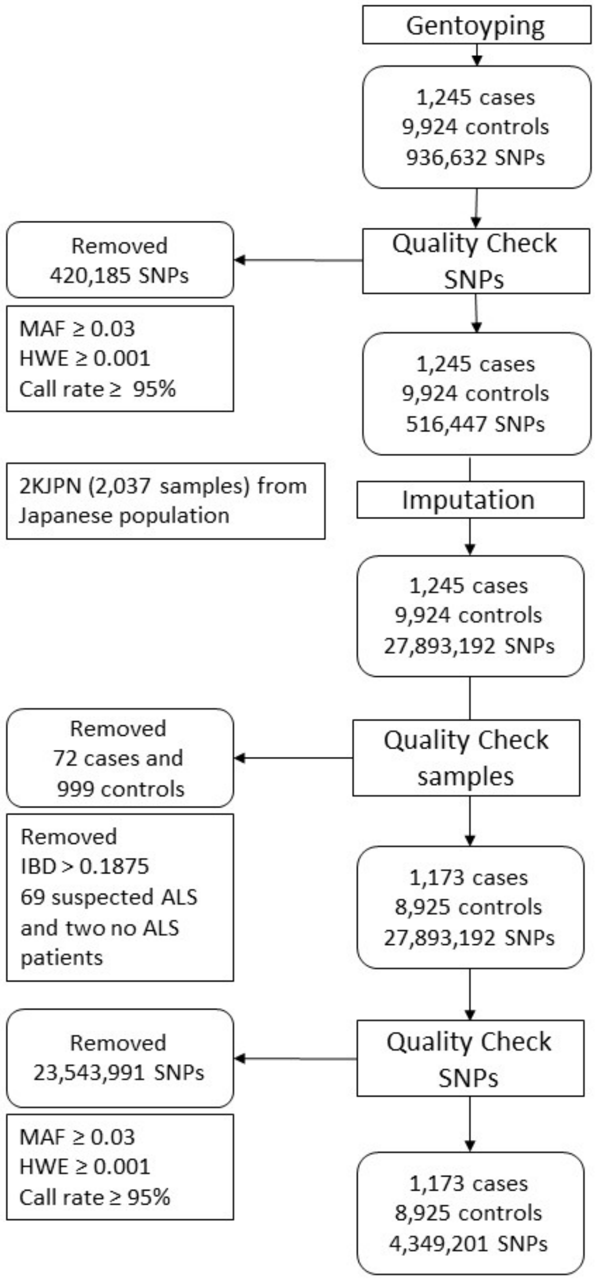

Supplementary Figure 1. The genotyping and imputation workflow for Japanese ALS patients and controls.

For the combined genotyped dataset from 1,245 cases and 9,924 controls, we selected SNPs with minor allele frequency ≥ 0.03, Hardy–Weinberg equilibrium ≥ 0.001, and genotype call rate ≥ 95%. This step retained 516,447 SNPs from 936,632 SNPs. The qualified genotype dataset was imputed with the 2,037 whole-genome reference panel (2KJPN) from TMM using Impute 4. The target individuals themselves for the imputation were not included in the 2KJPN reference panel. Finally, we

constructed the imputed dataset with 1,245 cases and 9,924 controls from 27,893,192 SNPs. We excluded 69 suspected ALS patients and two non-ALS patients from 1,245 cases. Samples that were identical-by-descent (>0.1875) among 1,174 cases and 9,924 controls were removed. Finally, 1,173 cases and 8,925 controls were used for the GWAS. For GWAS, we selected 4,349,201 SNPs with minor allele frequency ≥ 0.03, Hardy–Weinberg equilibrium ≥ 0.001, and genotype call rate ≥ 95%. We conducted logistic regression analysis with the principal components 1–20 from the principal component analysis as covariates using Plink (version 1.90b5.1).

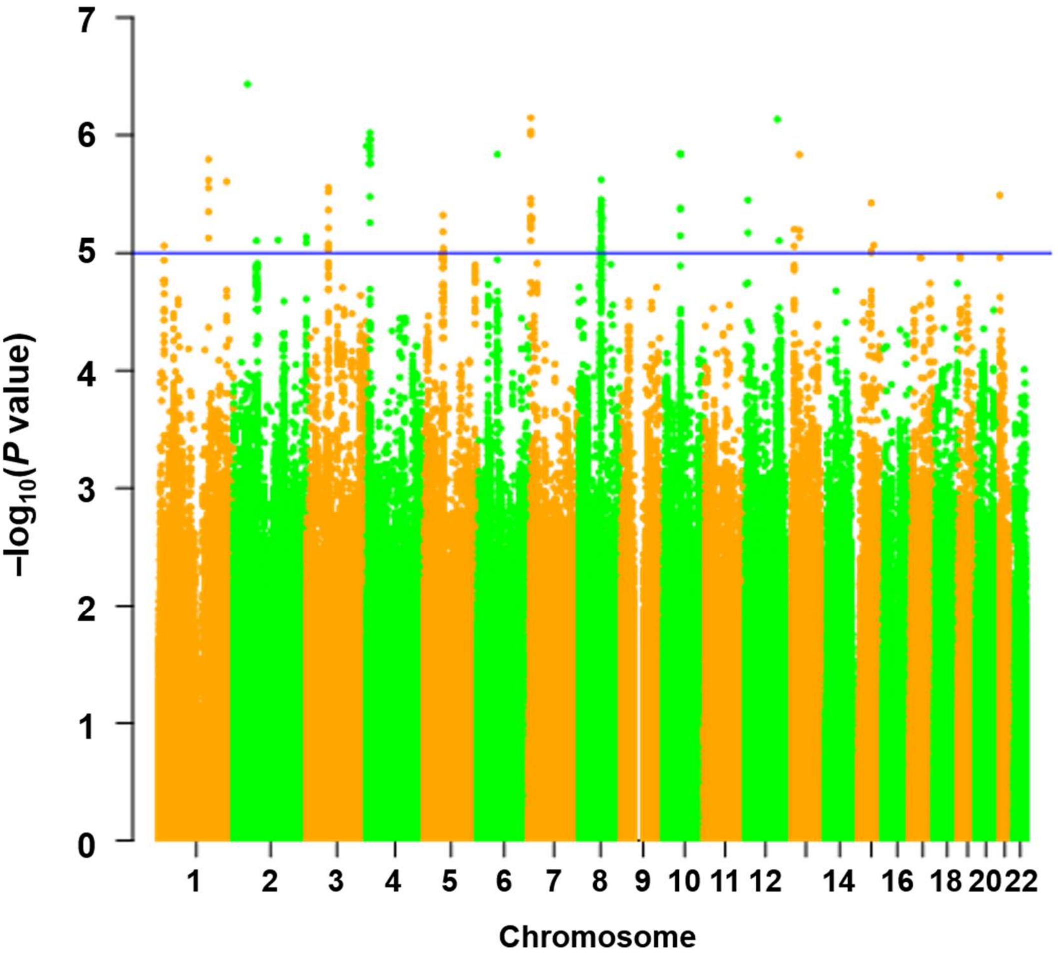

Supplementary Figure 2. Manhattan plot of the GWAS results of a Japanese (JaCALS) cohort. We conducted a genome-wide association study in a Japanese sample of 1,173 sporadic ALS cases and 8,925 controls. The 56 SNPs passed the condition with $p < 5.0 \times 10^{-6}$, while no individual SNPs passed the genome-wide significant p-value threshold of 5.0×10^{-8}. The blue line indicates the suggestive threshold of $p = 5.0 \times 10^{-6}$.

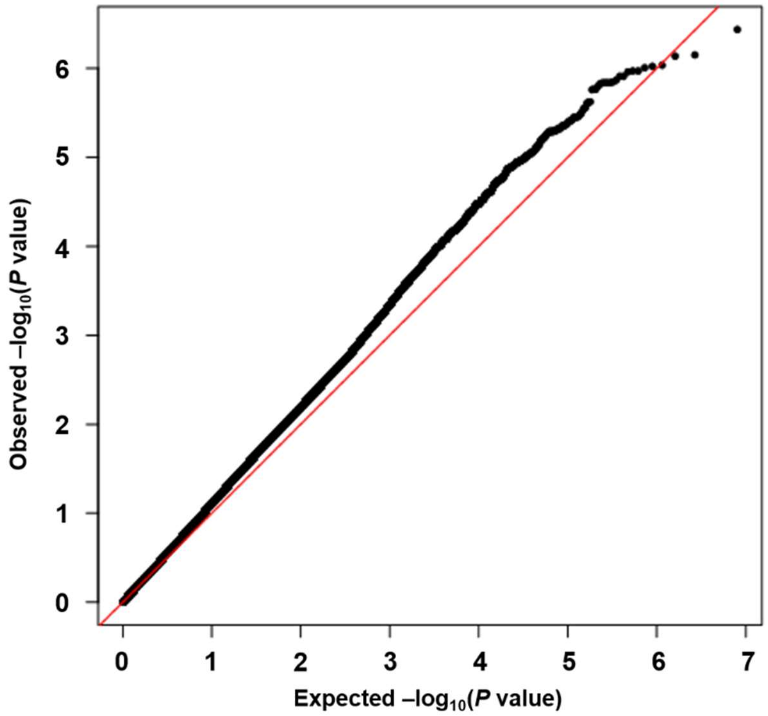

Supplementary Figure 3. Q–Q plots of the association –log10(P) between SNPs and ALS by GWAS of a Japanese (JaCALS) cohort.

a. rs58854276

Study	N(case)	N(control)	OR (95%CI)	p
**GWAS**				
European	20,806	59,804	1.07 (1.04-1.10)	1.06e-06
Japanese	1,173	8,925	1.18 (1.07-1.31)	1.62e-03
**Meta-analysis (GWAS)**	**21,979**	**68,729**	**1.08 (1.07-1.10)**	**2.97e-08**
**Replication**				
Chinese	1,234	2,850	1.20 (1.08-1.36)	1.46e-04
Japanese 2nd	707	971	0.98 (0.85-1.12)	7.62e-01
**Meta-analysis (replication)**	**1,941**	**3,821**	**1.11 (1.06-1.16)**	**1.56e-02**
**Meta-analysis (overall)**	**23,920**	**72,550**	**1.08 (1.07-1.10)**	**1.79e-09**

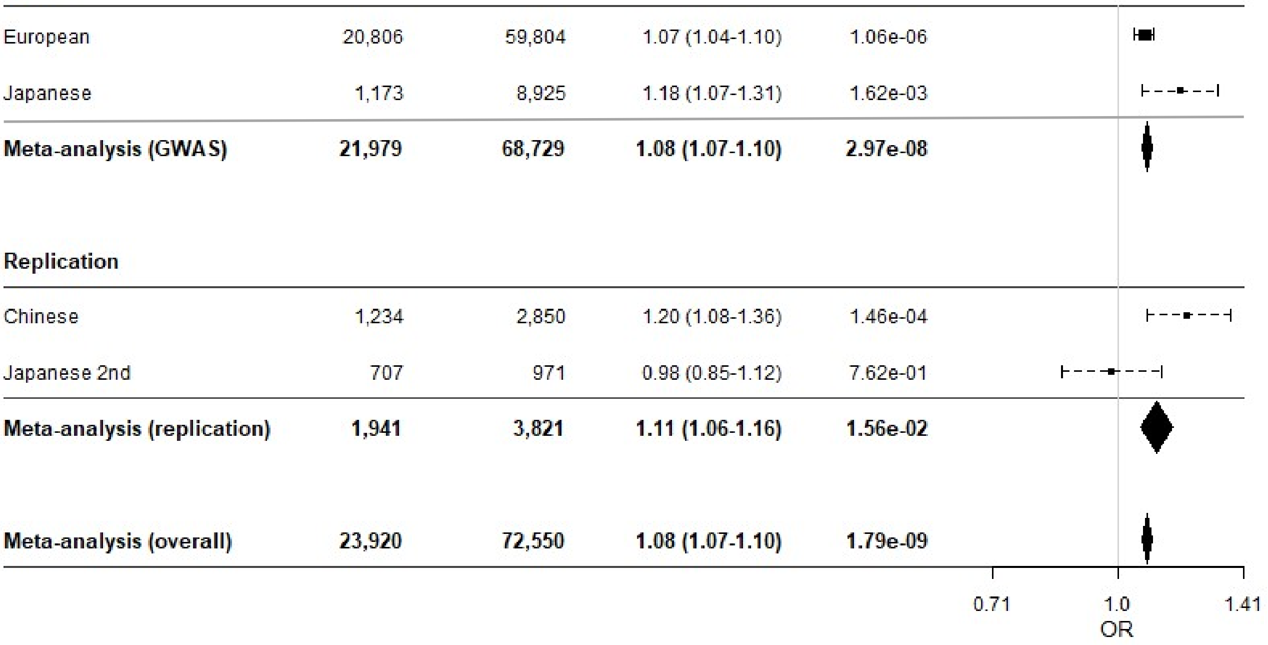

b. rs11195948

Study	N(case)	N(control)	OR (95%CI)	p
**GWAS**				
European	20,806	59,804	1.07 (1.05-1.10)	9.14e-07
Japanese	1,173	8,925	1.16 (1.05-1.29)	4.18e-03
**Meta-analysis (GWAS)**	**21,979**	**68,729**	**1.08 (1.06-1.09)**	**3.99e-08**
**Replication**				
Chinese	1,234	2,850	1.20 (1.10-1.29)	2.09e-04
Japanese 2nd	707	971	0.96 (0.84-1.10)	5.54e-01
**Meta-analysis (replication)**	**1,941**	**3,821**	**1.14 (1.10-1.18)**	**1.82e-04**
**Meta-analysis (overall)**	**23,920**	**72,550**	**1.09 (1.07-1.10)**	**8.20e-11**

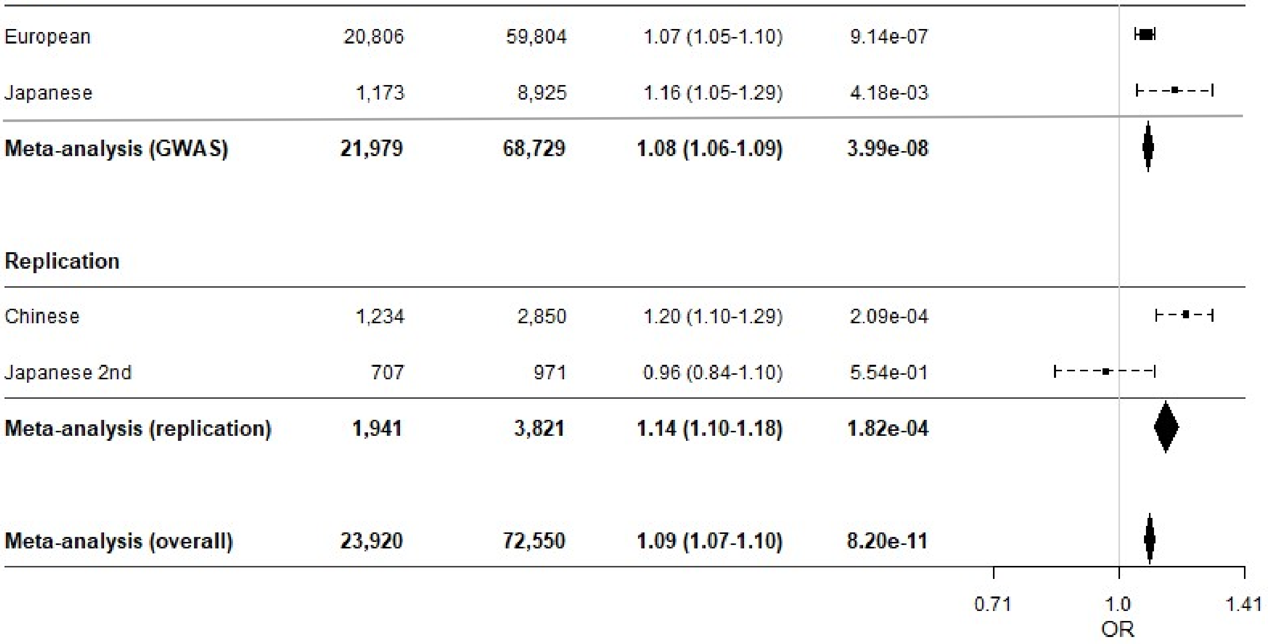

Supplementary Figure 4. Forest plots showing the effects of rs58854276 and rs11195948 in *ACSL5* in each cohort and meta-analysis.

Forest plots showing the effects of **a** rs58854276, and **b** rs11195948 on ALS in each cohort and meta-analysis.

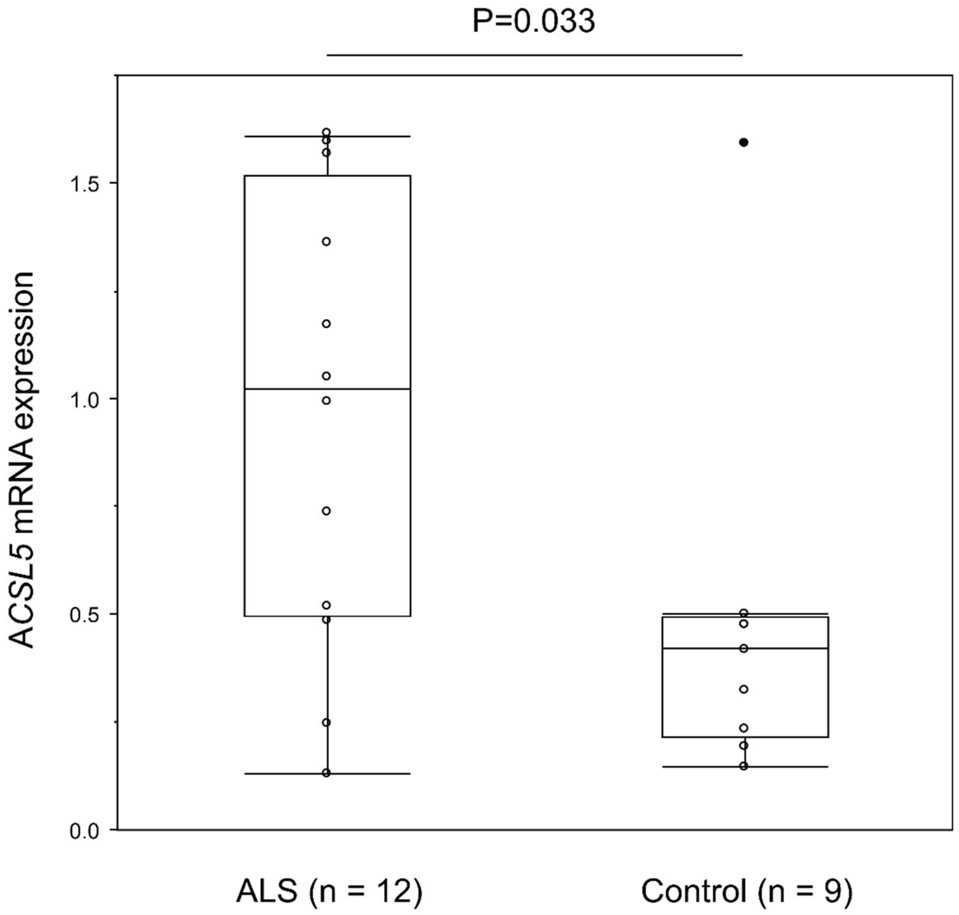

Supplementary Figure 5. Box plot of *ACSL5* mRNA expression between sporadic ALS and controls.

The mRNA expression of spinal motor neurons isolated by laser-capture microdissection in 12 sporadic ALS patients and 9 controls were originally observed by Batra et al[1,2]. We obtained the RNA seq data from the NCBI Gene Expression Omnibus (accession number GSE76220), and analyzed the mRNA expression of *ACSL5*. The *ACSL5* mRNA expression is possibly higher in sporadic ALS than in controls (p-value = 0.033 with Mann–Whitney U test). Circles represent individual data points and one black circle represents outlier. The bottom and the top of the box indicates the interquartile range (the 25th and 75th percentiles) and the line represents the median. The whiskers under and over the box correspond to the minimum and maximum values.

Supplementary Reference

1. Batra, R. *et al.* Gene expression signatures of sporadic ALS motor neuron populations. Preprint at *BioRxiv*, https://www.biorxiv.org/content/10.1101/038448v2 (2016).

2. Krach, F. *et al.* Transcriptome-pathology correlation identifies interplay between TDP-43 and the expression of its kinase CK1E in sporadic ALS. *Acta Neuropathol.* **136**, 405-423 (2018).

Supplementary Table 1. Clinical characteristics of the patients used for the gene expression analysis of *ACSL5*.

Genotype of rs3736947	AA	AC	CC	P value
N	20	20	20	
Age (years) (mean±SD)	62.9±7.4	61.7±10.5	61.7±7.8	0.873
Men/Women (N)	10/10	15/5	15/5	0.153

N, number of patients.

Comparison of age was performed using the one-way analysis of variance test, and comparison of sex-ratio among the three genotype groups was done using the $\chi 2$ test.

Supplementary Table 2. Primer sequences for quantitative real-time PCR

Gene Symbol	Name	Forword primer	Reverse primer
ACSL5	Acyl-CoA Synthetase Long Chain Family Member 5	5′ - AAGGCATTGGTGCTGATAGG -3′	5′ - TCAGGTCTTCTGGGCTAGGA -3
B2M	Beta-2-microglobulin	5′ - CTGAAGCTGACAGCATTCGG -3′	5′ - GTCAACTTCAATGTCGGATGGATG -3′
