# Supplementary material for: A multi-ethnic meta-analysis identifies novel genes, including ACSL5, associated with amyotrophic lateral sclerosis
Source: Commun Biol. 2020 Sep 23;3:526. doi: 10.1038/s42003-020-01251-2 (PMC7511394; doi:10.1038/s42003-020-01251-2)
Supplement: Supplementary file 2 — Description of Additional Supplementary Files [file 42003_2020_1251_MOESM2_ESM.pdf]

Description of additional supplementary items

Supplementary Data 1. Supplementary Data 1. Summary of the SNPs with a suggestive threshold of $p = 5 \times 10^{-6}$ in the GWAS of a Japanese (JaCALS) cohort.

Supplementary Data 2. Summary of the SNPs with a suggestive threshold of $p = 5 \times 10^{-6}$ in the GWAS of the meta-analysis between European and Japanese (JaCALS) cohorts.

Supplementary Data 3. Summary of the SNPs with a significant threshold of $p = 5 \times 10^{-8}$ in the GWAS study of the meta-analysis among European, Japanese (JaCALS), and Chinese cohorts.

Supplementary Data 4. The data of expression of *ACSL5* mRNA in LCLs from ALS patients with each genotype of rs3736947.

Supplementary Data 5. Summary of genes with a suggestive threshold of $p = 2.85 \times 10^{-4}$ in the multi-ethnic meta-analysis of gene-based association analysis among European, Japanese (JaCALS), and Chinese cohorts.
